# Public Trust in Different Sources of Information: Gaps in Rural Residents and Cancer Patients

**DOI:** 10.3390/healthcare13060640

**Published:** 2025-03-15

**Authors:** Wei-Chen Lee, Emily M. Kim, Elizabeth A. Nemirovski, Sagar Kamprath, Meredith C. Masel, Darpan I. Patel

**Affiliations:** 1 Department of Family Medicine, University of Texas Medical Branch, Galveston, TX 77555, USA; 2 John Sealy School of Medicine, University of Texas Medical Branch, Galveston, TX 77555, USA; emmkim@utmb.edu (E.M.K.); eanemiro@utmb.edu (E.A.N.); 3 Family Medicine Program, Houston Methodist Hospital, Houston, TX 77030, USA; sagar232@gmail.com; 4 School of Public and Population Health, University of Texas Medical Branch, Galveston, TX 77555, USA; mcmasel@utmb.edu; 5 School of Nursing, University of Texas Medical Branch, Galveston, TX 77555, USA; daipatel@utmb.edu

**Keywords:** source of information, health information-seeking behavior, cancer, rural

## Abstract

Background/Objectives: Understanding health information-seeking behavior is critical in providing effective interventions and improving quality of life for patients, especially those facing complex diagnoses like cancer. The purpose of this study is to understand rural–urban differences in trust levels for various information sources and how trust may differ by cancer status (no cancer, newly diagnosed, survived for six and more years). Methods: We examined 5775 responses from the 2022 Health Information National Trends Survey^®^. Using the component analysis, eight sources of information were classified into three domains: structured (doctor, government, scientist, and charity), less structured (family and religion), and semi-structured (health system and social media). Respondents answered questions on a scale of 1–4. Weighted linear regression models were constructed to examine trust level in three domains by rural residency and cancer status, while adjusting for demographic and socioeconomic status. Results: Urban patients reported higher trust in more structured sources of information (2.999 > 2.873, *p* = 0.005) whereas rural counterparts reported higher trust in less structured sources of information (2.241 > 2.153, *p* = 0.012). After adjusting for covariates, urban respondents with cancer are more likely to trust doctors (Coeff. = 0.163, *p* < 0.001) than those without cancer. Rural respondents with cancer are less likely to trust charities (Coeff. = −0.357, *p* < 0.01) and scientists (Coeff. = −0.374, *p* < 0.05) than rural respondents without cancer. Conclusions: Newly diagnosed cancer patients in rural areas are less likely to trust structured sources of information even after adjusting for all covariates. Additional studies about misinformation and disinformation being channeled through less structured sources of information are needed to prevent any delay in care among cancer patients, especially rural patients who are more likely to access these sources of information.

## 1. Introduction

Health information-seeking behavior (HISB) has been conceptualized as a key coping strategy in health-promotive activities and psychosocial adjustment to illness [1]. Individuals may search for health information regarding the symptoms, diagnoses, and treatments of different diseases or more general information about weight loss, healthy diets, or vitamin and mineral supplements. Understanding HISB is essential for designing educational and supportive interventions to promote self-management knowledge and skills, especially for vulnerable populations like those living in rural areas, demonstrating the need to evaluate rural–urban health disparities [2]. Sources of information may include various channels, such as family members, healthcare providers, newspapers, or the internet [3]. Health care providers are the most frequently used source of information, but in the digital age, online HISB has surged among consumers and patients due to lower cost and wider accessibility [4,5].

According to the 2022 Cancer Statistics, there were an estimated 1.9 million new cancer diagnoses in 2022 and more than 18.1 million cancer survivors in the US [6,7]. The growing number of cancer survivors is a testimony to the success of early detection and more effective treatment, but survivors from rural areas may experience less access to either preventative or curative intervention, in comparison to their urban counterparts. For instance, a study using the 2007–2019 National Provider Identifier data found that nearly two-thirds of counties in the US had no oncologist primarily based there and that 12% of counties also lacked an oncologist in an adjacent county [8]. Meanwhile, local primary care providers were found to lack experience in treating survivors of certain types of cancers and could not find specialists to refer patients to [9]. Rural patients and caregivers, therefore, have expressed less trust in local providers because their local doctors could not provide adequate resources and support. Medical staff located in metropolitan treatment centers are not sufficiently aware of unique needs of rural patients which can vary greatly from their urban patients [10]. Compared to rural patients with low health literacy, urban patients with low health literacy may have greater exposure to accessible health information through a variety of sources, including billboards, health clinics, and community organizations [11]. As a result, for rural cancer patients, there is an unmet need for information on treatment plans and side effects and for information on coping and transitioning back to daily life [12]. While the literature has pointed out HISB differences between rural and urban areas, investigation by cancer status has not been conducted yet. A recent review concluded that both cancer- and rural-relevant content on a national scale is limited because (1) many surveys did not include geographic data, (2) the definition of rural area is inconsistent across different surveys, and (3) the final sample size is too small to yield meaningful results [13]. In response to the demand for understanding and assessing trends in cancer-related communication, the National Cancer Institute (NCI) launched an annual data collection, called Health Information National Trends Survey (HINTS), in 1997 [14]. The HINTS data not only enable policy makers to better understand HISB, but also cancer-related knowledge, attitudes, and behavior for the non-institutionalized adult population in the US. A PubMed search between 2009 and 2024 revealed only 40 articles studying rural disparities using the HINTS data. Publications consistently demonstrate the gap between urban and rural access to health information and electronic devices as well as lower involvement in healthy behaviors. For instance, Hu and colleagues reported that individuals living in non-urban areas are less likely to access their electronic health information (adjusted Odds Ratio [aOR] = 0.6) [15]. Around half of the 40 articles examined the belief, knowledge, and behaviors of individuals with cancer or focused on cancer-related information. However, only one study investigated the sources of information for rural and urban adults, and it did not stratify population by their cancer status [16]. As such, more research using the HINTS data to study HISB in both cancer- and rural-contexts is needed.

Health information-seeking behaviors can vary significantly within cancer patients based on factors such as type of cancer and stage of diagnosis, emphasizing how each patient’s condition is unique. Although there is limited research on the reasons why cancer type may influence the information-seeking style, relationships have been found between cancer types and HISB. For instance, according to a study done on 731 cancer patients of various types, female breast cancer patients had higher odds of having active health information-seeking behavior. In the same study, patients, independent of cancer type, were generally found to be more active in their health-seeking behavior prior to treatment compared to afterward, demonstrating that not only type of cancer, but stage of cancer diagnosis can affect a patient’s HISB. This relationship was specifically noted within lung cancer patients of both sexes and male genitourinary cancer patients. A potential reason for this correlation of active information-seeking during the pretreatment period may be that patients seek increased education about treatment options when they are the most involved in discussions about their care. Moreover, a poorer prognosis was found to lead to patients being more passive in seeking information on their condition compared to patients with a more favorable prognosis [17]. A 2023 systematic review found that patients with a diagnosis of cancer with a higher stage were less likely to have online information-seeking behaviors in 12% of the studies analyzed. In comparison, patients who had just received a cancer diagnosis were more likely to display health-seeking behaviors in 16% of studies [18]. These differences highlight the variability in health information-seeking behaviors within the cancer population and the need for further research to tailor future interventions to unique needs of individual patients.

Improving health communication is critical so that people with cancer can easily understand and act on health information. Alleviating rural disparities in access to health information is also critical to facilitate positive health behavior, prevention, treatment, and provider recommendations. The purpose of this study was to understand the sources of information trusted by non-institutionalized adults in the US and their confidence levels in eight sources of information. This study further examined whether having cancer or not is associated with their confidence in different sources of information. In our hypothesis, the survivors who self-reported a recent diagnosis may be more proactive in seeking health information. The findings may provide insight into which information sources cancer patients and cancer survivors in rural areas trust and may inform interventions to better deliver accurate and timely health information to people in rural areas.

## 2. Materials and Methods

Data for this study were drawn from the 2022 Health Information National Trends Survey^®^ (HINTS), commissioned by the National Cancer Institute to collect nationally representative data about the American public’s use of cancer-related information [19]. The HINTS has identified changing communication trends and practices, provided information about how cancer risks are perceived, and investigated cancer information access and usage almost every year since 2003. The de-identified data are publicly available on the HINTS website and the 2022 data comprise a population-based sample of 6252 respondents.

This study included adults aged 18 and above who could make informed consent without a surrogate or legal representative. After removing 477 respondents (7.6%) who missed reporting their age and gender, the final sample consisted of 5775 individual responses. Based on the 2013 Rural–Urban Continuum Codes (RUC2013) available in the HINTS data, we divided the sample into two groups. The urban group contains individuals who live in counties in metro areas with more than 250,000 people (RUC2013 values 1–3), and the rural group contains individuals who live in counties with fewer than 250,000 people (RUC2013 value 4–9). In total, there were 758 (13.3%) rural respondents and 5017 (86.7%) urban respondents in this 2022 HINTS dataset, which is close to the national statistics (85%) [20].

The primary outcome was assessed with the question, “In general, how much would you trust information about cancer from each of the following: doctor, family or friends, government health agencies, charitable organizations, religious organizations and leaders, or scientists?” The answers include 1 = Not at all, 2 = A little, 3 = Some, and 4 = A lot. Meanwhile, respondents were asked if they use information from social media to make decisions about their health. The answers include 1 = Strongly Disagree, 2 = Somewhat Disagree, 3 = Somewhat Agree, and 4 = Strongly Agree. Finally, respondents were asked, “How much do you trust the healthcare system (for example, hospitals, pharmacies, and other organizations involved in health care)?” The answers also range from 1 = Not at all to 4 = A lot.

Because respondents may simultaneously trust multiple resources, we adopted the principal component analysis (PCA) to reduce a set of intercorrelated variables into a few dimensions. Table 1 revealed that eight sources of information could be classified into three components to explain at least 39% of variance. The first component is related to more structured sources of information, including doctors, government, charity organizations, and scientists. The second component is related to the group that may not use structured, systematically collected, or evidence-based information, such as family and religion. The third component is considered semi-structured. Given that the original questions in the HINTS did not specify types of social media and health systems, any materials from them could be either structured (e.g., a post from a professional society with scientific citations) or unstructured (e.g., a testimony post from a patient case). Therefore, we created the semi-structured component for sources including social media and health systems. After we finalized three dimensions, we created the second set of the study outcomes, the mean score for each component. The score of the first component was generated by dividing the sum of the four scores (doctor, government, charity, scientist) by four, and vice versa. The final score for the first component Table 1 was 3.08, and 2.22 for both second and third components.

The primary predictor is the time since cancer diagnosis based on when survivors were diagnosed with cancer and age when completing the HINTS survey. For analysis, this variable was coded as 0 = no cancer, 1 = 6 years or more after diagnosis (“survivors”), and 2 = 0–5 years after diagnosis (“patients”).

Covariates included age, gender, race, education, financial conditions, insurance status, and number of chronic conditions. Because the HINTS data do not have detailed geographic information to reflect consumer price index, we used the question, “Which one of these comes closest to your own feelings about your household’s income?” to determine a respondent’s financial condition. The variable is coded as 0 = finding it difficult or very difficult on present income, 1 = getting by, and 2 = living comfortably.

In addition to cancer, each respondent was asked to check what comorbid condition(s) they have, including diabetes, hypertension, heart disease, lung disease, depression, and obesity. Each response could be either 0 (no condition) or 1 (yes, has this condition). We summarized the scores of six conditions and divided them into three categories from 0 = no condition, 1 = one condition, and 2 = more than one condition.

To better reflect the complex sampling frame and acquire the national level of estimates, the HINTS data offer sampling stratum, weight, and primary sampling unit (PSU) variables for investigators. HINTS data use jackknife replicate weights to ensure the computation of correct variance estimates. In this study, we used the personal level of weight for all analyses and by applying the weights, our study sample represents opinions from 238,971,225 U.S. adults. More information on definition of sample weight and methodology to calculate variance estimate can be found in the HINTS user manual [19].

Statistical analyses were performed using STATA v18 (College Station, TX, USA). Descriptive analysis provided weighted frequency, mean, standard errors, percentage, and 95% Confidence Interval (95% CI) to describe the distributions of demographics, socioeconomic status, and confidence in 8 sources of information by three survivor levels and two geographic areas. Rural–urban differences in level of trust were stratified by 8 sources of information and by three major components, despite the cancer status. Chi-squared analysis and paired t-test assessed the relationship between survivor level and outcome for rural and urban respondents. Multivariate analysis further examined the relationship while controlling for all covariates. We have tested other variables, such as marital status, exercise, and smoking, and we did not find any significant relationship with the source of information or cancer survivorship. Therefore, we did not adjust our multivariate models for these factors. Predictors were found to be statistically significant with *p* < 0.05.

## 3. Results

### 3.1. Study Demographics

Table 2 and Table 3 divided the sample by their rural/urban residence and their cancer status (no cancer, cancer survivors over 6+ years, and cancer patients with recent diagnosis within the past 0–5 years). For either rural or urban respondents, each table showed the demographic differences by three cancer statuses to examine if those most recently diagnosed might be different from those without cancer or have survived for 6 years and more. Table 2 shows that compared to cancer patients (diagnosed in 0–5 years) and survivors (diagnosed more than 6+ years), those without cancer and living in rural areas were about 15 years younger (*p* < 0.001). Among male respondents, 5.1% were recently diagnosed with cancers (0–5 years), which is higher than the percentage of female respondents (3.3%) (*p* = 0.0179). There are no differences in distributions of race, education, financial status, and number of chronic conditions between cancer patients and individuals without cancer. Around 13.2% of insured respondents have cancer (0–5 years or 6+ years), significantly higher than those without insurance (*p* = 0.0146).

Table 3 shows that in urban areas, the average age is significantly higher for those diagnosed with cancer for 6 or more years than those without cancer (68.7 > 46.9 years old) (*p* < 0.001). There are also significant differences in distributions of gender, race, education, finance, insurance status, and number of chronic conditions between cancer patients, survivors, and non-cancer respondents. It is notable that there is a higher percentage of AAPI (2.1% > 1.6%) and uninsured (1.7% > 1.3%) people who were recently diagnosed with cancer than the percentages of people who are cancer survivors, whereas other racial groups or people with insurance had a higher percentage of cancer survivors (6+ years) than recent cancer patients (0–5 years).

A comparison between rural and urban no-cancer groups shows a statistically significant difference in age (*p* < 0.001), with the rural group older than the urban one (51.4 in Table 2 > 46.9 in Table 3). However, there is no rural–urban difference in age for those diagnosed in the past 5 years (*p* = 0.308) or more than 6+ years (*p* = 0.126). Unlike rural respondents, a higher proportion of females in urban areas were newly diagnosed with cancer than males (Table 3, 3.9% > 2.5%, *p* = 0.0108). In addition, being non-Hispanic white (*p* < 0.001), having higher educational attainment (*p* = 0.0474), living comfortably on present income (*p* = 0.0014), being covered by any kind of health insurance (*p* = 0.0011), and having more than one chronic condition (*p* < 0.001) are positively related to being a cancer patient or survivor.

### 3.2. Levels of Trust in Information Sources

**Figure 1:**
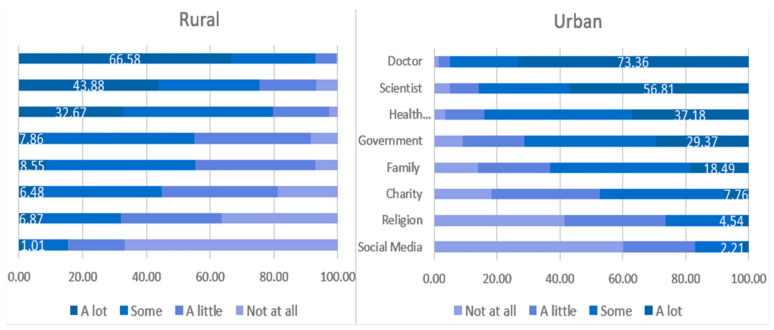
Rural–urban differences in level of trust by eight sources of information.

Rural and urban respondents differed in level of trust for eight sources of information. Figure 1 used a blue gradient spectrum to show the trust level from dark blue (trust a lot) to light blue (not trust at all). For both rural and urban respondents, the most reliable source is doctor (66.58% in rural and 73.36% in urban), and the least reliable source is social media (Figure 1, 1.01% in rural and 2.21% in urban). However, while 29.37% of urban respondents trusted the government “a lot,” the corresponding number is only 7.86% among rural respondents.

**Figure 2:**
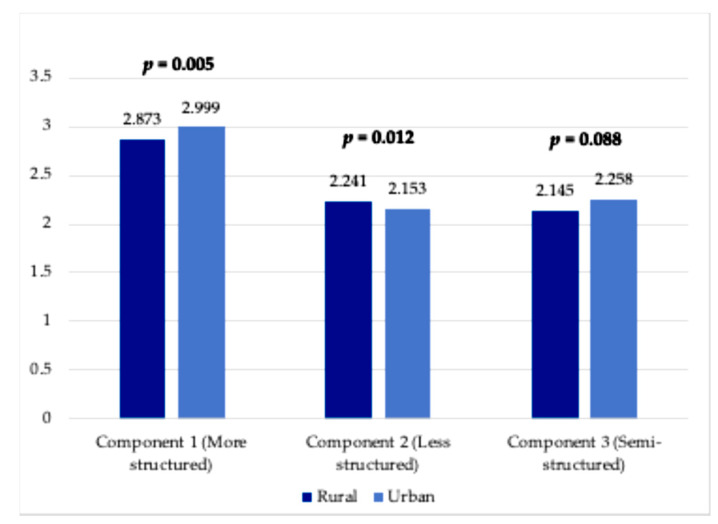
Rural–urban differences in level of trust by three components.

There are significant rural–urban differences by component (Figure 2). Figure 2 used the mean score of their trust levels (from 1 = not trust at all to 4 = trust a lot) and used dark blue to present rural respondents’ scores while light blue for urban respondents. Urban respondents are more likely to trust information from doctor, charity, government, and scientist (2.999 > 2.873, *p* = 0.005), but rural respondents are more likely to trust information from family and religion (2.241 > 2.153, *p* = 0.012). Although urban respondents have a higher confidence level in social media and health systems than rural respondents, the difference is not statistically significant (2.258 > 2.145, *p* = 0.088).

Table 4 illustrates the unadjusted associations between cancer status and trust level in eight sources of information. A higher score indicates a higher level of trust in the information provided by that specific source. Regardless of cancer status and rurality, most respondents trust information from their doctors (avg. range [3.58, 3.85]). On the contrary, social media is the least reliable source (avg. range [1.36–1.60]). Cancer patients (0–5 years) in rural areas self-reported that they trust information from family (mean score: 2.57 > 2.49) or religion (1.90 > 1.87) more than their urban counterparts, who trust information from doctors (3.85 > 3.62), government (2.88 > 2.47), charities (2.36 > 1.92), scientists (3.32 > 2.71), social media (1.54 > 1.36), and health systems (3.33 > 3.24) more than those living in rural areas. Finally, rural respondents who were diagnosed with cancer for 6 or more years (survivors) had a higher level of trust in all sources than newly diagnosed people or non-cancer people except for social media.

Table 5 shows the adjusted association between level of trust and time from cancer diagnosis using the group without cancer as the reference for rural and urban respondents. After controlling for seven covariates, cancer patients or survivors in urban areas are more likely than those without cancer to trust structured and semi-structured resources like doctors (Coeff. = 0.163, *p* < 0.0015) but less likely to trust less-structured resources such as family (Coeff. = −0.131, *p* < 0.05) and religion (Coeff. = −0.200, *p* < 0.001). On the other hand, cancer patients or survivors in rural areas are less likely to trust charity organizations (Coeff. = −0.357, *p* < 0.01) and scientists (Coeff. = −0.374, *p* < 0.05) than those without cancer. There is no significant difference in trust in government, social media, or health systems related to cancer status for both urban and rural respondents.

## 4. Discussion

### 4.1. Overall Research Findings

This study examined the makeup of rural and urban cancer patients and survivors and their subsequent differences in trust in various health information sources, highlighting three important findings. First, the descriptive analysis showed that the age of rural respondents without cancer is higher than their urban counterparts, indicating that living in urban areas might be related to early onset of cancer. Additionally, the average age between rural newly diagnosed cancer patients and rural survivors is not much different, but the difference in average age is seven years for urban respondents, indicating that urban respondents with cancer had a longer life span. Second, our rural–urban comparisons pointed out that a doctor is the source with the highest trust level by both rural and urban cancer patients, and social media has the least trust level. However, urban respondents had more trust in highly structured resources (doctor, government, charity, and scientist), whereas rural respondents had more trust in less structured resources of information (family and religion). This finding remains the same even after adjusting for demographics and socioeconomic conditions. Finally, our comparison of trust level by cancer status showed that cancer survivors in rural areas have a higher trust in all resources except for social media than recently diagnosed patients or non-cancer respondents, but there is no significant pattern by cancer status among urban respondents.

### 4.2. Discussion About Population Makeup

At a population level, we found differences in the demographic makeup of rural vs. urban populations with no diagnosis of cancer, a recent cancer diagnosis, and a long-term cancer diagnosis. Incidence rates for cancer are highest for older populations with a median age of 66, as it takes time for DNA damage to incur cancerous mutations [21]. However, our study found that the average age of urban respondents newly diagnosed with cancer is below the national median. In contrast, the average age of urban respondents who have survived cancer for 6 and more years is older than their rural counterparts. Some studies have found that living in an urban setting may increase risk of developing cancer, while increased geographical proximity to medical care and cancer screenings enhanced survivorship, which may explain the older average age of individuals with a cancer diagnosis of 6+ years from urban areas [22,23].

Our data showed that most individuals with a diagnosis of cancer had insurance coverage, regardless of whether they lived in rural or urban areas. Continued health needs of cancer patients often include maintenance treatment, treatment for cancer-related side-effects, monitoring for progression or recurrence, or mental health treatment, which results in increased utilization of the healthcare system and thereby necessitates insurance coverage to afford services [24]. Given that cancer is typically diagnosed at older ages, increased insurance rates among those with cancer may be due to more cancer patients being on Medicare, which begins at age 65 [25]. This correlates closely with the average age of 65.4 for newly diagnosed rural cancer patients in our study. It is possible that new access to insurance through Medicare increases access to healthcare for rural populations, especially for non-expansion states, incentivizing them to seek out delayed health screenings or care for pre-existing symptoms and leading to new-found cancer diagnoses.

### 4.3. Rural–Urban Differences in Trust Level

This study found that cancer patients in rural and urban areas may have different levels of trust in the information sources they seek. Both urban and rural groups overall report the highest amounts of trust in their doctors. Cancer patients report higher levels of trust in doctors than those without cancer, and this effect was most pronounced for urban respondents with a recent cancer diagnosis. While doctors have been and remain the primary trusted source of health information, many cancer patients report unmet information needs [26,27,28]. This implies they seek out other sources of health information, such as family, peers, online support groups, or the internet.

Overall, urban respondents reported significantly higher trust in “more structured” sources of information, while rural respondents reported significantly higher trust in two less structured sources of information. This finding may be concerning, as the quality of cancer-related health information may differ greatly between structured and unstructured sources [29,30]. The difference in source of information is most stark when comparing trust in government sources of information for urban vs. rural respondents, with only 7.86% of rural respondents reporting “a lot” of trust in government information (vs. 29.37% for urban). It could be a public health crisis when rural populations are less likely to trust in government-sponsored information sources, such as interventions for control of infection and disease. As a result, rural individuals may face difficulty making accurately informed decisions regarding cancer care due to barriers in accessing high-quality health information compared to their urban counterparts [11]. This deficit has the potential to widen the disparity in adverse health outcomes, including poor survival rates among rural populations. Governments and scientists should therefore strive to disseminate their work on a lay-person level and involve community partners.

Increased reliance on unstructured sources of information may also create additional financial burdens on both individuals and the healthcare system. While asking family members or religious guides for health information does not imply substantial costs over the short term, inaccurate information from such sources could conceivably lead to long-term costs due to postponed doctor visits or poor health choices [5]. Our study also noticed that social media is the least trusted source despite rurality or cancer status. Internet use has potential to narrow the gap in accessing information that can be used to improve quality of life and survivorship for individuals with cancer, regardless of geographical residence [31]. According to our research finding, future work can tailor where high-quality information is channeled through social media to improve how cancer patients can participate in HISB. Doctors can also direct rural cancer patients and survivors to reliable internet resources, which could mitigate the information gap among those with limited access to specialists. Health communication professionals should outreach rural individuals and communities and design appropriate health information materials, taking local norms and dialects into consideration [32].

### 4.4. Differences in Trust Level by Cancer Status—No Cancer, Cancer Survivors, and Cancer Patients

Patients’ level of trust in various information sources differed by cancer status in this study. Compared to the respondents without cancer, survivors and those with newly diagnosed cancer have higher trust in doctors and health systems, perhaps because individuals are required to place life-changing decisions into the hands of their doctors. One Danish study, however, found that while the majority of patients’ trust in their general practitioner was high both before and after cancer diagnosis, their confidence decreased after cancer diagnosis from 88.4% to 80.0% as a result of doctors’ delays in diagnosis, so missing the opportunity for early treatments [33]. Therefore, more research is necessary to understand the progression of a cancer patient’s trust in providers and health systems after diagnosis.

This study also found that cancer patients living in rural areas had less trust in charity organizations and scientists, although rural survivors appear to have more trust in all resources except for social media. A diagnosis of cancer may increase self-efficacy in seeking healthcare and willingness to maintain personal health [34], but the ability to do so may be hindered by access to reliable guiding information. While we hypothesized that individuals with newly diagnosed cancer may have a higher demand for information, it is interesting to see a higher trust level in rural cancer survivors. Former studies indicate that as time since diagnosis increases, the level of mental distress experienced by patients tends to decrease and the amount of health perception increases [35]. This could explain how trust level might vary by time from diagnosis and the importance of establishing trust in the earlier stage of cancer treatment for rural populations when they experience the most amount of psychological distress. More studies to identify why rural cancer survivors are less likely to trust in charity organizations and scientists are needed to tailor information to build trust in these structured sources.

### 4.5. Influence of Social Media

After adjusting for demographics and socioeconomic factors, our study found that urban cancer patients and survivors had more trust in social media, which is the opposite to the association before we controlled covariates. This finding is aligned with the literature that age and socioeconomic conditions are driving factors of internet use [36] and another finding that cancer patients who use the internet and social media for cancer information reported greater internet confidence [37]. The internet has been found to be the most frequently cited source of information for cancer patients—more than healthcare providers, brochures, and cancer organizations—and plays a fundamental role in access to health information for patients [38]. With the large role that the internet plays in providing and exchanging health information, there is a risk of patients obtaining inaccurate and harmful health information or using it incorrectly. It is worrisome that only 30.4% of patients who reported using the internet for health information discussed it with their providers [38]. One study that analyzed cancer-related content on social media found that 67% of the content was accurate, while 19% was inaccurate. This highlights how social media can provide both helpful, accurate information as well as inaccurate and potentially harmful content [39]. Another study that examined available online information for pancreatic cancer found that the online resources that were found to be accurate were often at a high reading level, limiting the accessibility of accurate information for vulnerable populations. This study also found that nonprofit, academic and government websites were the most accurate [40], highlighting the importance of research on the types of information sources patients may trust. The COVID-19 pandemic has only worsened these concerns. The pandemic has intensified the reliance on social media and the internet for health information [41], while also exacerbating the existing health disparities among vulnerable populations [42]. The worsened health outcomes in vulnerable groups, coupled with the increase in reliance on potentially unchecked social media information, emphasizes the growing need for research on interventions focused on online information-seeking behaviors. Additional studies to examine what online information cancer patients and survivors receive, how they use it, and why they are reluctant to discuss with their providers are critical to improving patient safety and outcomes.

### 4.6. Study Limitations

It is important to acknowledge the limitations of this study. First, the survey questions did not offer sufficient definitions of social media platforms, types of health systems, or charity organizations. We used the component analysis to classify eight sources into three categories, and these three domains may change if we survey a different population. Next, considering this is a cross-sectional study, we could not determine the causal relationship between social determinants, cancer status, and trust in sources of health information. Third, the HINTS data are focused on understand the general public’s knowledge, sources of health information, and trust levels in those sources. Other factors such as a person’s ability to self-care and outcomes such as delay in care or use of supplements are not collected. Additional qualitative studies might be helpful to illustrate the association between trust levels and health outcomes.

## 5. Conclusions

This study contributes new information on how likely each subgroup (rural/urban; no cancer, long-term survivor, recently diagnosed patient) is to trust the information they are given. This study shows that levels of trust in information sources vary based on level of structure for urban and rural cancer patients, with rural patients reporting higher levels of trust in unstructured information sources than urban patients. Cancer patients are a unique population, as the disease is complex and course of treatment can vary greatly among specific diagnoses, stage, and population, requiring informed decision making. Understanding what sources cancer patients and survivors are most likely to trust is critical to meeting their health needs and supporting HISB in a positive manner.

## Figures and Tables

**Table 1:** Principal component analysis for eight sources of information.

Corresponding Factor Loading	Highly Structured	Less Structured	Others (Semi-Structured)
Doctor	**0.4252**	−0.2800	0.2312
Family	0.2659	**0.3952**	0.1927
Government	**0.4828**	−0.1428	−0.1832
Charity	**0.4227**	0.3267	−0.3656
Religion	0.2336	**0.5800**	−0.0581
Scientist	**0.4189**	−0.2895	−0.2804
Social Media	0.0402	0.3866	**0.5150**
Health System	0.3242	−0.2620	**0.6285**

**Table 2:** Demographics and socioeconomic status by time to cancer diagnosis—rural.

Rural	No Cancer	Diagnosed More Than 6+ Years	Diagnosed in 0–5 Years	*p*-Value
**Age (mean, std.)**	51.4 (0.97)	66.5 (1.85)	65.4 (2.37)	<0.001
**Gender (%, 95%CI)**				0.0179
Female (*n* = 14,370,060)	85.2 (81.1, 88.6)	11.4 (8.3, 15.6)	3.3 (2.0, 5.6)	
Male (*n* = 15,359,510))	89.4 (85.5, 92.3)	5.5 (3.6, 8.4)	5.1 (3.1, 8.4)	
**Race (%, 95%CI)**				0.3824
Non-Hispanic White (*n* = 22,782,307)	85.3 (82.3, 88.0)	9.8 (7.6, 12.6)	4.8 (3.2, 7.1)	
Non-Hispanic Black (*n* = 2,166,475)	92.5 (83.1, 96.9)	3.7 (1.5, 8.7)	3.8 (0.9, 15.2)	
Hispanic (*n* = 1,796,938)	98.6 (93.3, 99.7)	0.0	1.4 (0.3, 6.7)	
Non-Hispanic AAPI ^‡^ (*n* = 595,623)	100.0	0.0	0.0	
Non-Hispanic Others (*n* = 2,388,228)	88.6 (69.7, 96.3)	9.4 (2.6, 29.1)	2.0 (0.4, 8.7)	
**Education (%, 95%CI)**				0.3509
Less than high school (*n* = 2,788,211)	93.2 (88.5, 96.1)	4.2 (2.1, 8.2)	2.6 (0.8, 8.1)	
12 years or completed high school (*n* = 9,010,681)	86.1 (79.9, 90.7)	10.1 (6.2, 16.0)	3.8 (1.9, 7.4)	
Some college (*n* = 10,968,997)	84.5 (77.8, 89.5)	9.8 (6.2, 15.2)	5.7 (3.0, 10.4)	
College graduate or higher (*n* = 6,961,681)	90.5 (85.5, 93.9)	6.4 (3.6, 11.3)	3.1 (1.7, 5.5)	
**Finance (%, 95%CI)**				0.0501
Difficulty (*n* = 6,181,737)	89.8 (83.1, 94.1)	7.4 (3.8, 13.8)	2.8 (1.1, 7.2)	
Doing ok (*n* = 12,343,207)	89.8 (85.7, 92.9)	5.6 (3.8, 8.3)	4.5 (2.7, 7.6)	
Comfortable (*n* = 11,204,625)	82.9 (77.8, 87.0)	12.5 (8.6, 17.8)	4.6 (2.9, 7.3)	
**Insurance (%, 95%CI)**				0.0146
Uninsured (*n* = 3,186,102)	99.0 (92.9, 99.9)	1.0 (0.1, 7.1)	0.0	
Insured (*n* = 26,543,468)	85.8 (83.0, 88.2)	9.5 (7.5, 12.0)	4.7 (3.2, 6.8)	
**Multiple Conditions (%, 95%CI)**				0.3161
0 Condition (*n* = 7,589,648)	91.7 (85.7, 95.3)	5.3 (2.4, 11.5)	3.0 (1.4, 6.2)	
1 Condition (*n* = 7,244,941)	86.4 (79.4, 91.3)	10.3 (6.0, 17.0)	3.3 (1.5, 7.2)	
More than one (*n* = 14,894,981)	85.3 (81.1, 88.8)	9.4 (6.7, 13.0)	5.3 (3.4, 8.1)	

‡: Asian-American and Pacific Islanders.

**Table 3:** Demographics and socioeconomic status by time to cancer diagnosis—urban.

Urban	No Cancer	Diagnosed More Than 6+ Years	Diagnosed in 0–5 Years	*p*-Value
**Age (mean, std.)**	46.9 (0.48)	68.7 (1.06)	61.0 (1.66)	<0.001
**Gender (%, 95%CI)**				0.0108
Female (*n* = 103,171,036)	89.3 (87.6, 90.8)	6.8 (5.8, 8.0)	3.9 (2.9, 5.2)	
Male (*n* = 106,070,620)	92.2 (90.7, 93.5)	5.3 (4.2, 6.6)	2.5 (1.9, 3.3)	
**Race (%, 95%CI)**				<0.001
Non-Hispanic White (*n* = 120,646,452)	87.2 (85.5, 88.8)	8.3 (7.2, 9.5)	4.4 (3.5, 5.6)	
Non-Hispanic Black (*n* = 26,690,458)	95.0 (93.2, 96.4)	3.4 (2.4, 4.8)	1.6 (0.9, 2.7)	
Hispanic (*n* = 35,709,702)	96.1 (94.5, 97.2)	2.9 (1.9, 4.4)	1.0 (0.6, 1.9)	
Non-Hispanic AAPI ^‡^ (*n* = 19,131,787)	96.3 (93.6, 98.0)	1.6 (0.8, 3.3)	2.1 (0.9, 4.5)	
Non-Hispanic Others (%*n* = 7,063,257)	91.5 (83.0, 95.9)	5.1 (2.2, 11.6)	3.4 (0.9, 11.8)	
**Education (%, 95%CI)**				0.0474
Less than high school (*n* = 15,186,940)	93.2 (89.1, 95.8)	3.5 (1.9, 6.2)	3.3 (1.8, 6.0)	
12 years or completed high school (*n* = 42,108,200)	92.9 (91.0, 94.4)	5.1 (3.8, 6.8)	2.0 (1.2, 3.3)	
Some college (*n* = 81,658,090)	90.2 (88.0, 92.0)	6.7 (5.3, 8.5)	3.1 (2.2, 4.5)	
College graduate or higher (*n* = 70,288,426)	89.5 (88.2, 90.7)	6.4 (5.3, 7.7)	4.1 (3.2, 5.2)	
**Finance (%, 95%CI)**				0.0014
Difficulty (*n* = 41,991,548)	93.6 (91.2, 95.4)	4.7 (3.2, 6.8)	1.7 (0.9, 3.3)	
Doing ok (*n* = 76,019,235)	92.1 (90.4, 93.5)	5.2 (4.2, 6.5)	2.7 (1.8, 4.0)	
Comfortable (*n* = 91,230,872)	88.2 (86.7, 89.7)	7.3 (6.2, 8.7)	4.4 (3.4, 5.8)	
**Insurance (%, 95%CI)**				0.0011
Uninsured (*n* = 22,928,607)	97.0 (94.1, 98.5)	1.3 (0.6, 2.9)	1.7 (0.6, 4.8)	
Insured (*n* = 186,313,049)	89.9 (88.7, 91.0)	6.6 (5.8, 7.6)	3.4 (2.7, 4.3)	
**Multiple Conditions (%, 95%CI)**				<0.001
0 Condition (*n* = 7,589,648)	91.7 (85.7, 95.3)	5.3 (2.4, 11.5)	3.0 (1.4, 6.2)	
1 Condition (*n* = 7,244,941)	86.4 (79.4, 91.3)	10.3 (6.0, 17.0)	3.3 (1.5, 7.2)	
More than one (*n* = 14,894,981)	85.3 (81.1, 88.8)	9.4 (6.7, 13.0)	5.3 (3.4, 8.1)	

‡: Asian-American and Pacific Islanders.

**Table 4:** Unadjusted association between trust level and time to cancer diagnosis by eight sources and two residency places.

Total = 238,971,225	No Cancer	Diagnosed More Than 6+ Years	Diagnosed in 0–5 Years
Mean (S.E.)	N = 215,739,175	N = 15,200,815	N = 8,031,236
Doctor			
Rural	3.58 (0.04)	3.64 (0.08)	3.62 (0.10)
Urban	3.66 (0.02)	3.74 (0.04)	3.85 (0.03)
Government			
Rural	2.69 (0.06)	2.61 (0.11)	2.47 (0.14)
Urban	2.91 (0.03)	2.95 (0.07)	2.88 (0.07)
Charity			
Rural	2.35 (0.06)	2.31 (0.10)	1.92 (0.13)
Urban	2.38 (0.03)	2.20 (0.06)	2.36 (0.08)
Scientist			
Rural	3.15 (0.05)	3.06 (0.12)	2.71 (0.17)
Urban	3.38 (0.02)	3.39 (0.07)	3.32 (0.08)
Component 1			
Rural	2.89 (0.03)	2.79 (0.07)	2.67 (0.10)
Urban	3.00 (0.02)	2.98 (0.05)	3.08 (0.05)
Family			
Rural	2.57 (0.04)	2.58 (0.13)	2.57 (0.14)
Urban	2.55 (0.02)	2.45 (0.05)	2.49 (0.06)
Religion			
Rural	2.02 (0.06)	2.13 (0.16)	1.90 (0.14)
Urban	1.90 (0.03)	1.74 (0.05)	1.87 (0.06)
Component 2			
Rural	2.24 (0.04)	2.24 (0.11)	2.24 (0.12)
Urban	2.16 (0.02)	2.01 (0.04)	2.14 (0.05)
Social media			
Rural	1.51 (0.05)	1.41 (0.11)	1.36 (0.14)
Urban	1.60 (0.04)	1.43 (0.06)	1.54 (0.09)
Health system			
Rural	3.08 (0.04)	3.23 (0.11)	3.24 (0.13)
Urban	3.16 (0.02)	3.32 (0.05)	3.33 (0.08)
Component 3			
Rural	2.15 (0.03)	2.15 (0.09)	2.09 (0.08)
Urban	2.26 (0.02)	2.16 (0.04)	2.28 (0.05)

**Table 5:** Adjusted association between trust level and time to cancer diagnosis by source and rurality.

Ref: No Cancer (Coefficient and Std.)	Diagnosed More Than 6+ Years	Diagnosed in 0–5 Years
Doctor		
Rural	0.053 (0.091)	0.013 (0.106)
Urban	0.063 (0.046)	0.163 (0.038) ***
Government		
Rural	−0.095 (0.127)	−0.212 (0.159)
Urban	0.050 (0.077)	−0.054 (0.078)
Charity		
Rural	−0.022 (0.108)	−0.357 (0.124) **
Urban	−0.037 (0.063)	0.114 (0.083)
Scientist		
Rural	−0.109 (0.149)	−0.374 (0.183) *
Urban	0.088 (0.064)	−0.038 (0.078)
Component 1		
Rural	−0.055 (0.081)	−0.166 (0.105)
Urban	0.062 (0.054)	0.100 (0.048) *
Family		
Rural	−0.025 (0.129)	0.022 (0.142)
Urban	−0.131 (0.060) *	−0.099 (0.065)
Religion		
Rural	0.131 (0.185)	−0.177 (0.170)
Urban	−0.200 (0.061) ***	−0.0254 (0.071)
Component 2		
Rural	0.025 (0.119)	0.010 (0.128)
Urban	−0.129 (0.048) **	0.0003 (0.049)
Social media		
Rural	−0.107 (0.110)	−0.107 (0.133)
Urban	0.028 (0.084)	0.090 (0.082)
Health system		
Rural	0.072 (0.121)	0.097 (0.156)
Urban	0.020 (0.051)	0.046 (0.080)
Component 3		
Rural	0.045 (0.096)	0.022 (0.082)
Urban	0.032 (0.047)	0.084 (0.053)

*: *p* < 0.05, **: *p* < 0.01; ***: *p* < 0.001.

## Data Availability

The original data presented in the study are openly available online: https://hints.cancer.gov/data/default.aspx (accessed on 12 March 2025).

## Abbreviations

The following abbreviations are used in this manuscript:

HISB	Health information-seeking behavior
HINTS	Health In-formation National Trends Survey
NCI	National Cancer Institute
